# Supplementary material for: Adjusting 1.5 degree C climate change mitigation pathways in light of adverse new information
Source: Nat Commun. 2023 Aug 23;14:5117. doi: 10.1038/s41467-023-40673-4 (PMC10447517; doi:10.1038/s41467-023-40673-4)
Supplement: Supplementary file 1 — Supplementary Information [file 41467_2023_40673_MOESM1_ESM.pdf]

Adjusting 1.5 degree C climate change mitigation pathways in light of adverse new information: Supplementary Information

Ajay Gambhir, Shivika Mittal, Robin Lamboll, Dan Bernie, Laila Gohar, Adam Hawkes, Alexandre Köberle, Joeri Rogelj, Jason Lowe

S1 Modelling framework

We use an integrated assessment model (TIAM-Grantham[1]) which in this guise represents energy and industrial process emissions with a high degree of technological detail across all energy system sectors.

We constrain the scenarios with a CO_2 budget (for fossil fuel and industrial emissions, as well as land uise emissions) of 500 $GtCO_2$ from 1st January 2020, following IPCC AR6 WGI[2]. We then combine the resulting CO_2 emissions pathway derived from TIAM-Grantham with the CO_2 pathway for land use emissions (specifically, as derived from the median AFOLU pathway in the IPCC SR1.5 database[3] for all 1.5°C scenarios) with a non-CO_2 emissions pathway derived from the Silicone[4] in-filling model. The resulting pathways for all greenhouse gas emissions are then fed into the FaIR[5] simple climate model, to derive probabilistic temperature pathways.

This modelling set up allows us to vary technology deployment rate asssumptions, carbon budget levels, and underlying assumptions on energy demand, as required in our study design (Supplementary Figure 1). We use updated SSP2 assumptions as our underlying socio-economic driver, as described in Sognnaes et al. (2021)[1].

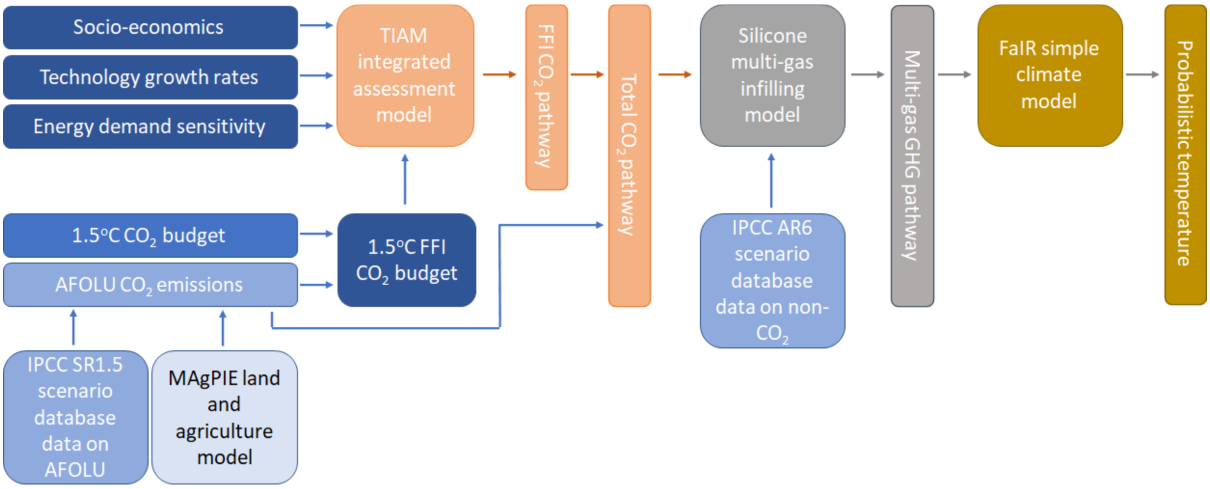

Supplementary Figure 1: Model and assumptions framework. Blue boxes designate input assumptions. TIAM model is used to derive fossil fuel and industrial (FFI) CO_2 pathways consistent with the specified carbon budget. AFOLU emissions are derived from either the IPCC scenario database or the MAgPIE model, depending on the specific scenario. IPCC scenario database also used to perform multi-gas infilling, using the Silicone model.

S2 Mitigation costs

The carbon price implications of the different scenarios are discussed in the main manuscript (and shown in Figure 8 in the main manuscript). In addition, we present in Supplementary Figure 2 the mitigation costs of each scenario. These are expressed as the change in welfare (producer plus consumer welfare) related to the energy system, when comparing each scenario with a "current policies" scenario, as developed and described in Sognnaes et al. (2021) [1].

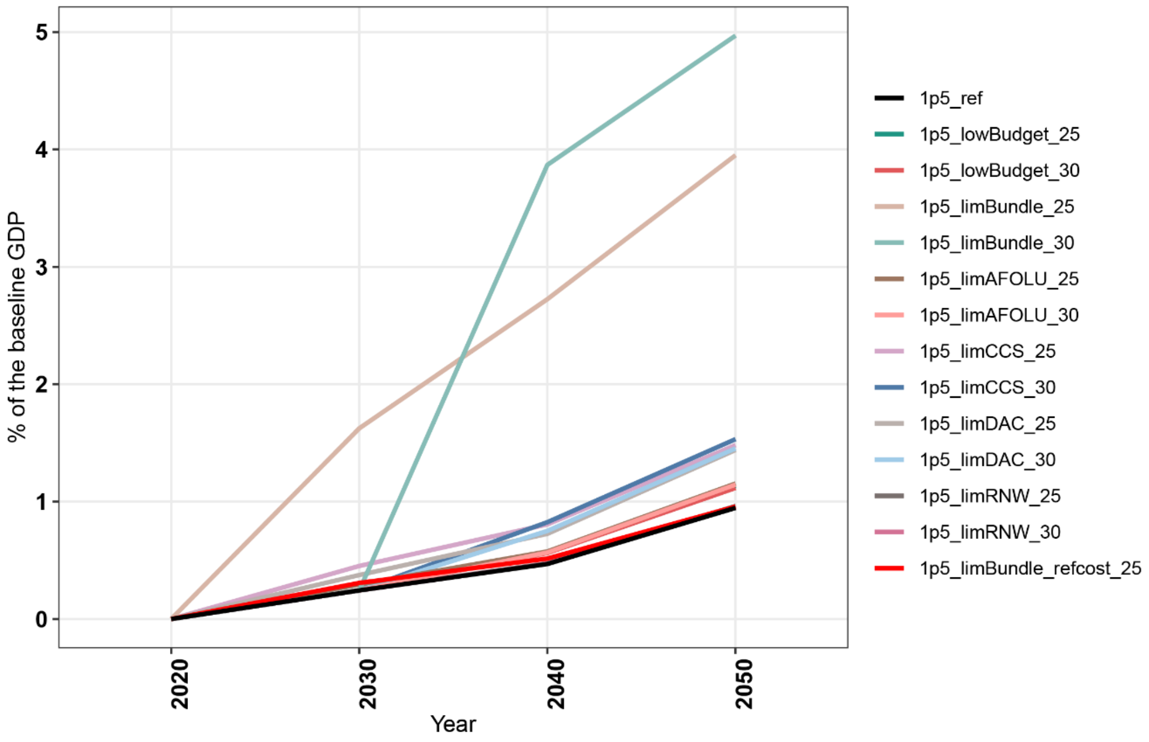

Supplementary Figure 2: Annual mitigation cost as share of baseline GDP. Scenarios as shown in the main manuscript, with one additional scenario, labelled "1p5_limBundle_refcost_25", simulated to provide a similar mitigation cost to the "1p5_ref" scenario, but with all adverse information (except for the carbon budget) arriving in 2023, leading to a course correction in 2025. This allows a minimum carbon budget of 1,100 GtCO2 whilst achieving the same mitigation cost pathway as the "1p5_ref scenario".

S3 Electricity as a share of final energy

Our scenarios have relatively low levels of electricity as a share of final energy, compared to the median across the IPCC AR6 database[6] of 1.5°C scenarios with no or low overshoot (i.e. the "C1" category). By 2050, our most electrified scenario (1p5_limBundleLD_30) has an electrification of ~50%, around the median of the IPCC AR6 database[6]. This could suggest that further electrification is possible as a course correction strategy.

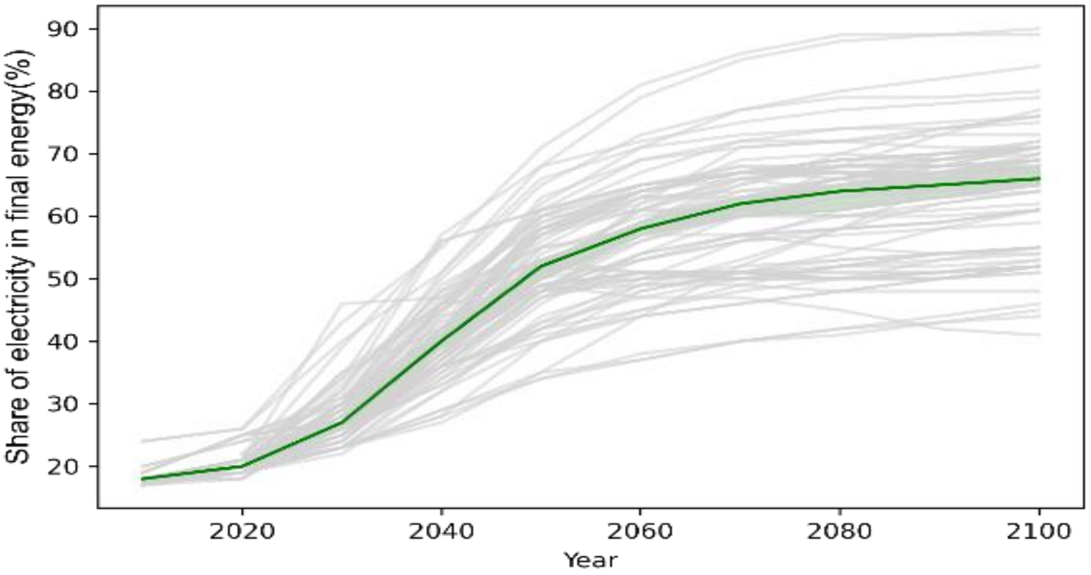

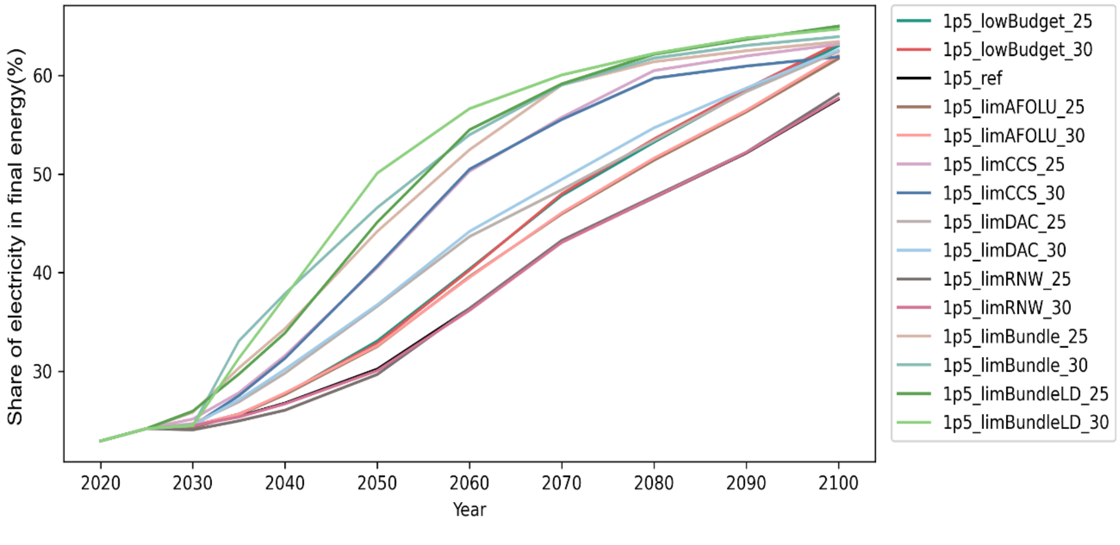

Supplementary Figure 3: Electricity as a share of final energy in 1.5°C scenarios. Top panel shows share of electricity in final energy in IPCC AR6 database of "C1" category (i.e. 1.5°C with no or low overshoot) scenarios, including median (green line), whilst bottom panel shows share of electricity in final energy from scenarios in this study.

S4 Carbon prices

The highest carbon price scenario, 1p5_limBundle_30, rises to ~$5,000/t$CO_2$ by 2050 and to over $20,000/t$CO_2$ by 2050, indicating severe economic feasibility concerns. This scenario is still, however, just within the range of no or low overshoot ("C1" category) scenarios in the IPCC AR6 scenarios database[6], as shown in Supplementary Figure 4.

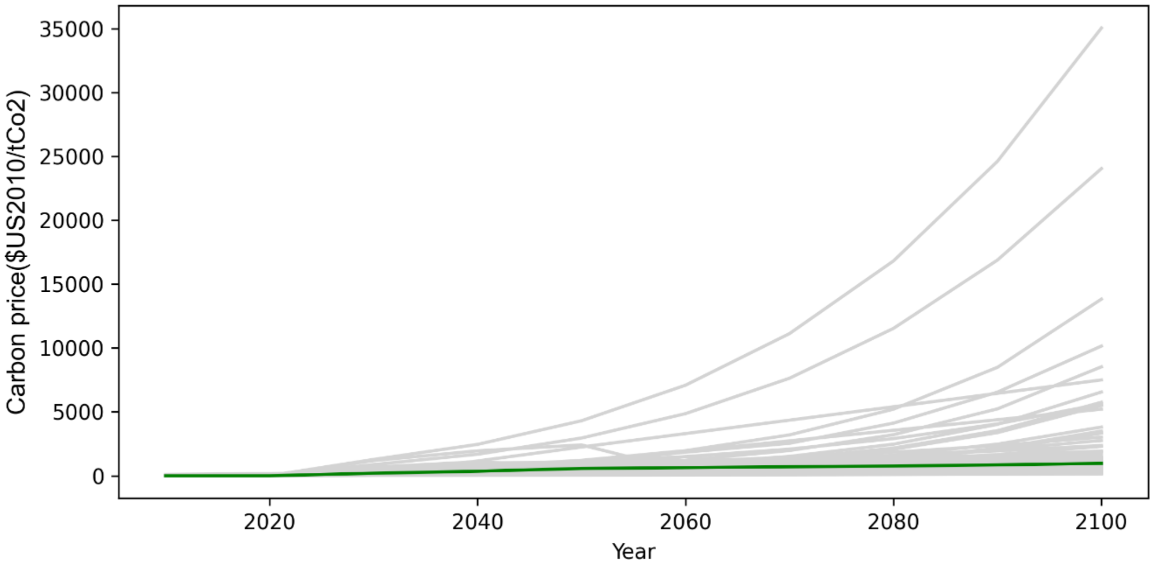

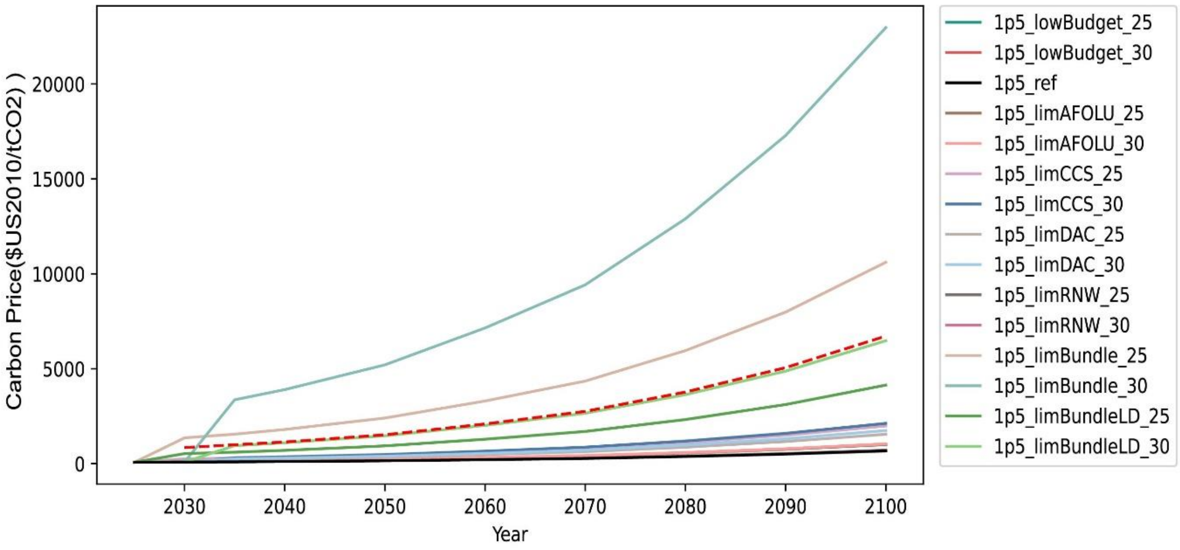

Supplementary Figure 4: Carbon prices in 1.5°C scenarios. Top panel shows carbon price in IPCC AR6 database of "C1" category (i.e. 1.5°C with no or low overshoot) scenarios, including median (green line), whilst bottom panel shows carbon prices from scenarios in this study. Bottom panel red dotted line is for a scenario in which all adverse information is assumed from the start of global mitigation action in 2023 (which means there is no mitigation pathway adjustment in this scenario).

S5 Temperature and climate implications of pathways

Our study's radiative forcing pathways, once non-CO_2 gases have been combined with the CO_2 emissions from the TIAM model (and where appropriate the MAgPIE model or the exogenous CO_2 assumptions from the IPCC SR1.5 database) follow a broadly similar pattern to the median pathway in the IPCC AR6 database[6] for no and low overshoot (i.e. category "C1") scenarios, as shown in Supplementary Figure 5.

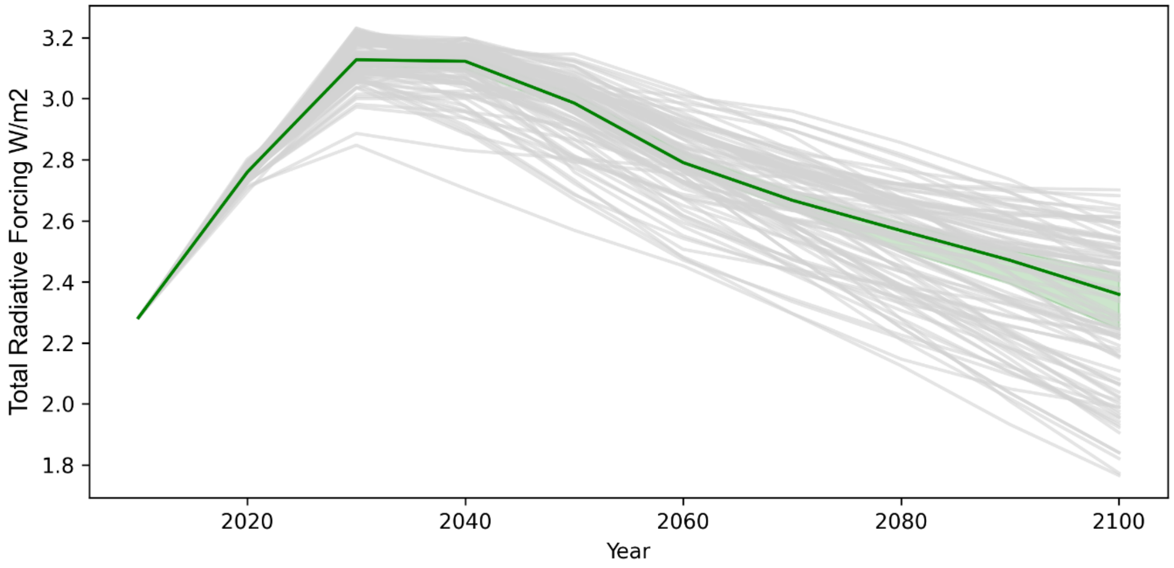

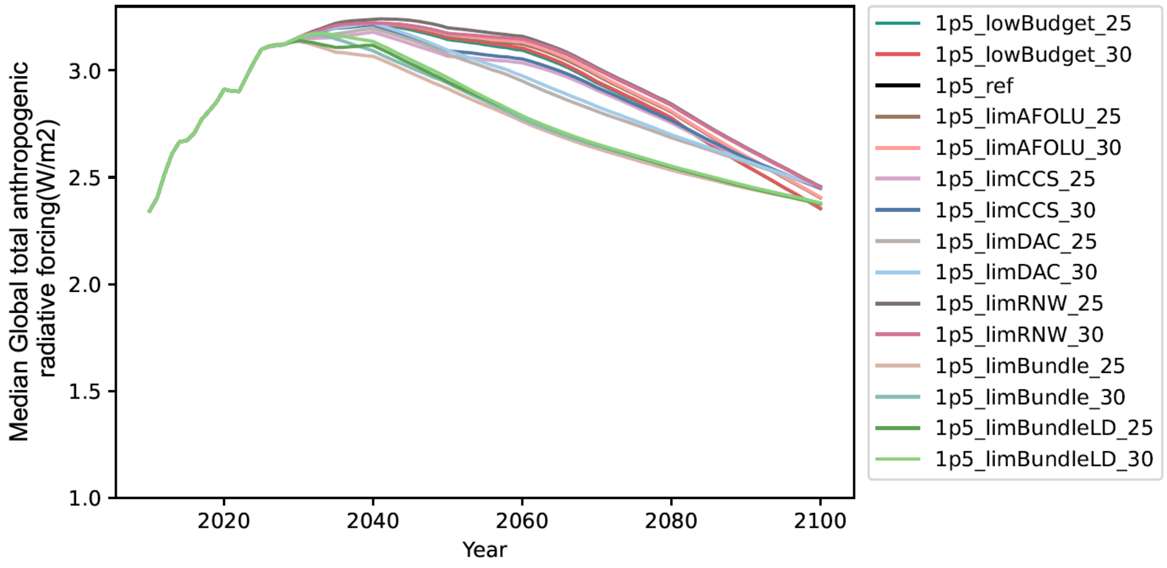

Supplementary Figure 5: Radiative forcing implications. Top panel shows forcing in IPCC AR6 database of "C1" category (i.e. 1.5°C with no or low overshoot) scenarios, including median as red line, whilst bottom panel shows forcing from scenarios in this study.

This results in similar temperature pathways, in most cases peaking below 1.6°C and falling back below 1.5°C by end of century (Supplementary Figure 6).

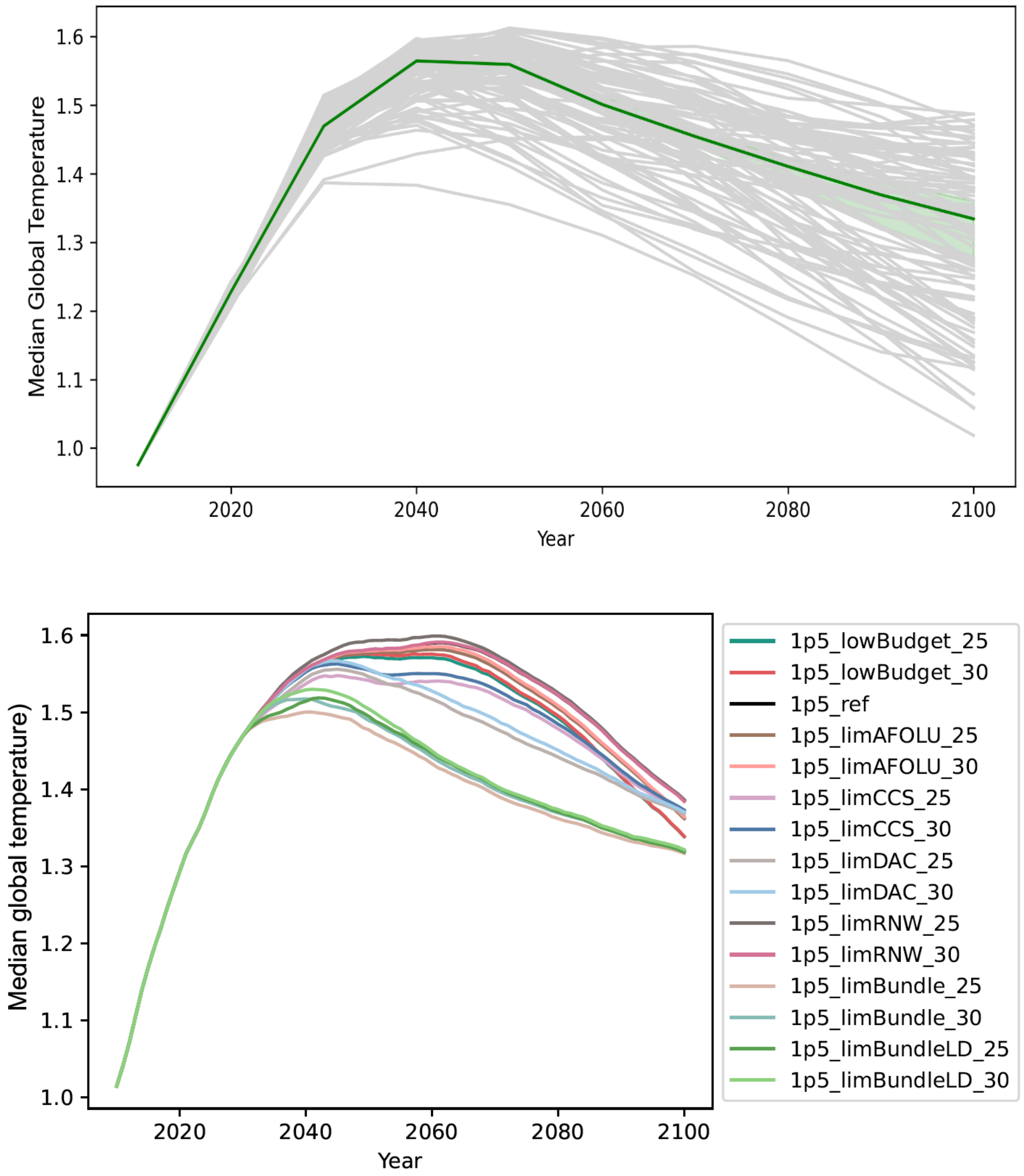

Supplementary Figure 6: Temperature implications. Top panel shows forcing in IPCC AR6 database of "C1" category (i.e. 1.5ºC with no or low overshoot) scenarios, including median as green line, whilst bottom panel shows forcing from scenarios in this study.

S6 Emissions pathways with different system discount rates

We show the mitigation pathways for discount rates ranging from 1-5% per year (Supplementary Figure 7). As demonstrated in other literature[7], lower discount rates see more drastic near-term emissions reductions and less overall net negative emissions in the latter half of the 21st century. This is demonstrated by their smaller 2020-2050 cumulative carbon emissions. All scenarios in which DAC and CCS growth rates become limited tend to have similar pathways, given the lack of availability of net negative emissions in the latter half of the 21st century. As such, the differences in cumulative CO_2 emissions in the period 2020-2050 for discount rate variants of these scenarios are far less marked than in the 1p5_ref scenarios.

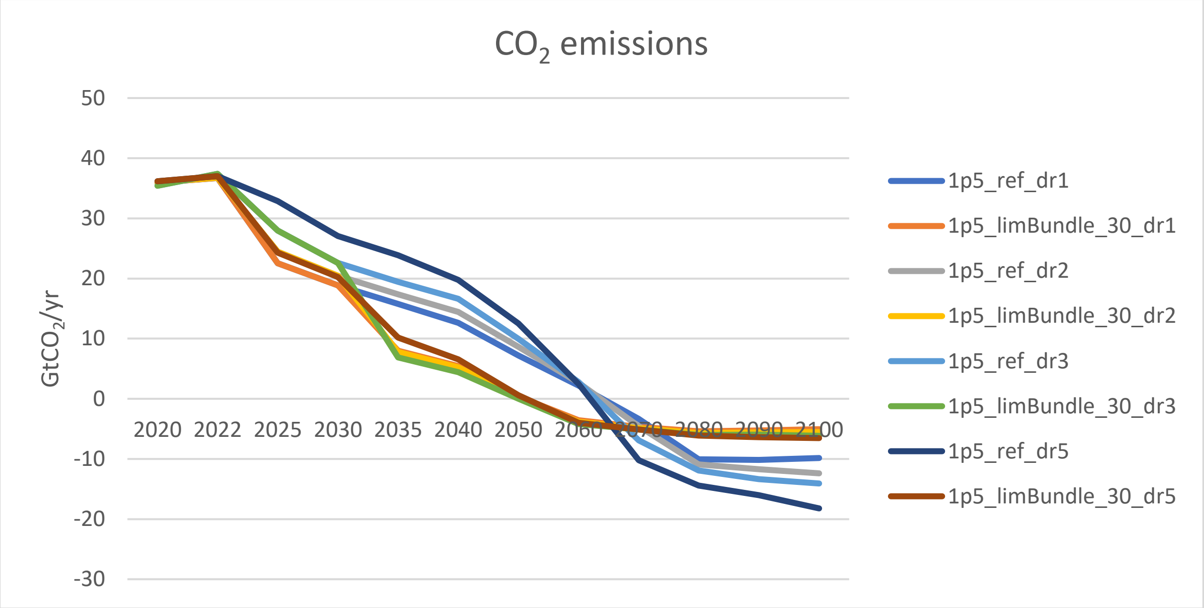

Scenario	Cumulative CO_2 (2020-2050)
1p5_ref_dr1	543
1p5_limBundle_30_dr1	413
1p5_ref_dr2	589
1p5_limBundle_30_dr2	425
1p5_ref_dr3	648
1p5_limBundle_30_dr3	439
1p5_ref_dr5	750
1p5_limBundle_30_dr5	448

Supplementary Figure 7: Mitigation pathways with different system discount rates. Top figure shows fossil fuel and industry global CO_2 emissions in 1p5_ref and 1p5_limBundle_30 scenarios, for a range of discount rates 1%, 2%, 3%, 5%. Bottom panel shows cumulative CO_2 from all emissions, including land use, for the period 2020-2050.

S7 Emissions pathways with different system discount rates

The TIAM-Grantham Integrated Assessment Model's outputs on key variables of interest in this study (for the 1p5_Ref scenario) are compared to those across the range of similar temperature pathway scenarios in the IPCC AR6 database[6] for no and low overshoot (i.e. category "C1") scenarios (Supplementary Figure 8). In general, the TIAM-Grantham model is not an outlier compared to the range, which is itself a limited representation of the future possibility space. However, the range is large, so model-specific results on any single technology do not represent the only possibility.

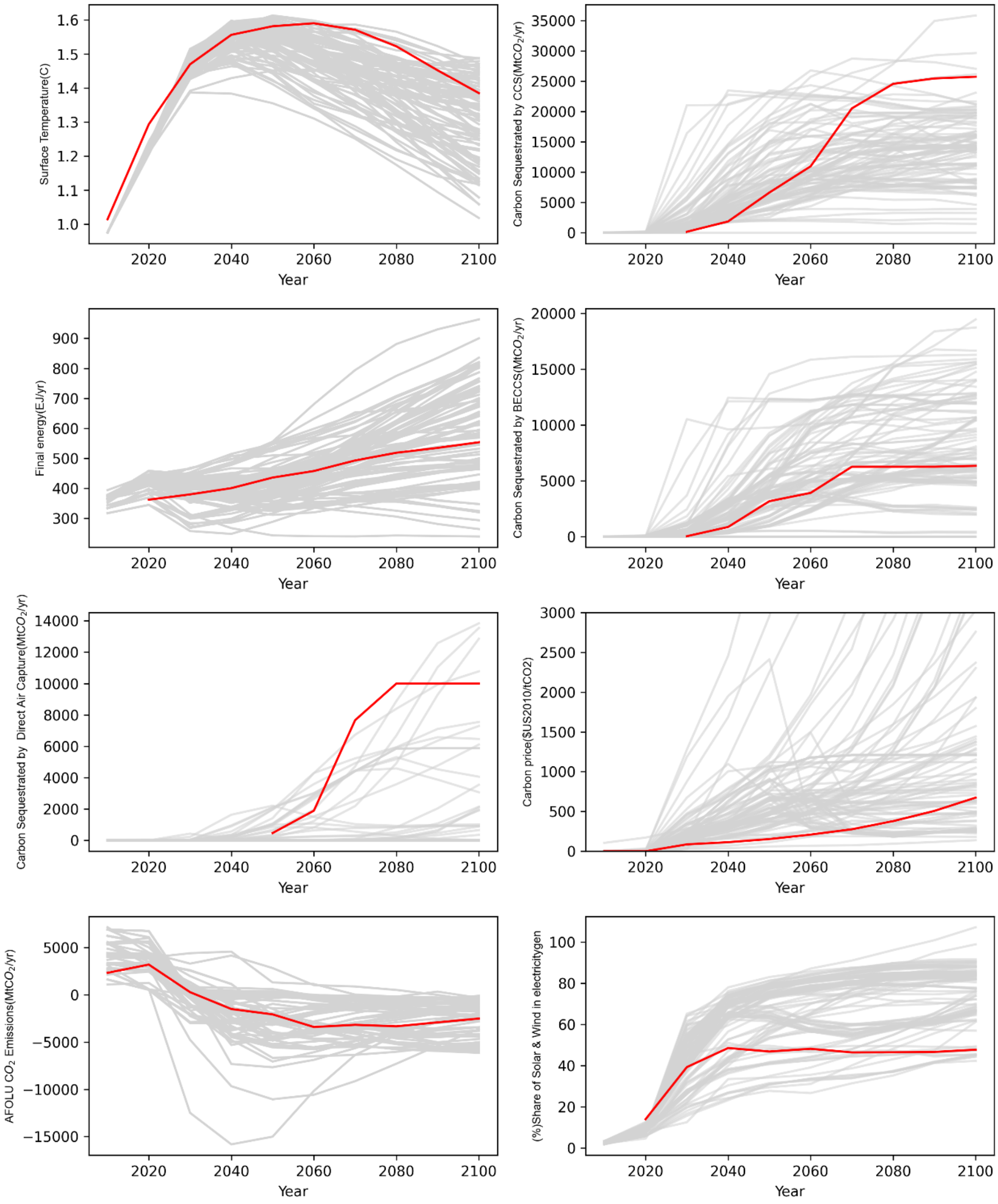

Supplementary Figure 8: Key variables in 1.5°C scenarios. IPCC AR6 database of "C1" category (i.e. 1.5°C with no or low overshoot) scenarios (grey lines) with overlay of this study's 1.5°C reference scenario (red line), for key variables analysed in this study.

Supplementary References

1. Sognnaes, I. *et al.* A multi-model analysis of long-term emissions and warming implications of current mitigation efforts. *Nat. Clim. Change* **11**, 1055–1062 (2021).
2. IPCC. *Climate Change 2021: The Physical Science Basis. Contribution of Working Group I to the Sixth Assessment Report of the Intergovernmental Panel on Climate Change [Masson-Delmotte, V., P. Zhai, A. Pirani, S. L. Connors, C. Péan, S. Berger, N. Caud, Y. Chen, L. Goldfarb, M. I. Gomis, M. Huang, K. Leitzell, E. Lonnoy, J. B. R. Matthews, T. K. Maycock, T. Waterfield, O. Yelekçi, R. Yu and B. Zhou (eds.)]*. (Cambridge University Press. In Press, 2021).
3. Huppmann, D., Rogelj, J., Krey, V., Kriegler, E. & Riahi, K. A new scenario resource for integrated 1.5 °C research. *Nat. Clim. Change* (2018) doi:10.1038/s41558-018-0317-4.
4. Lamboll, R. D., Nicholls, Z. R. J., Kikstra, J. S., Meinshausen, M. & Rogelj, J. Silicone v1.0.0: an open-source Python package for inferring missing emissions data for climate change research. *Geosci. Model Dev.* **13**, 5259–5275 (2020).
5. Smith, C. J. *et al.* FAIR v1.3: a simple emissions-based impulse response and carbon cycle model. *Geosci. Model Dev.* **11**, 2273–2297 (2018).
6. Byers, E. *et al.* AR6 Scenarios Database. (2022) doi:10.5281/zenodo.5886912.
7. Emmerling, J. *et al.* The role of the discount rate for emission pathways and negative emissions. *Environ. Res. Lett.* **14**, 104008 (2019).
